# Construction and analyses of the microRNA-target gene differential regulatory network in thyroid carcinoma

**DOI:** 10.1371/journal.pone.0178331

**Published:** 2017-06-01

**Authors:** Ying-ying Kang, Yang Liu, Ming-Li Wang, Min Guo, Yan Wang, Zhi-Feng Cheng

**Affiliations:** 1 Department of Endocrinology, Fourth Affiliated Hospital of Harbin Medical University, Harbin, China; 2 Clinical laboratory, Fourth Affiliated Hospital of Harbin Medical University, Harbin, China; Universitat des Saarlandes, GERMANY

## Abstract

Thyroid-carcinoma (THCA) is the most common malignancy with an increasing incidence. Recent evidence has emphasized the role of microRNA (miRNA) in THCA. However, knowledge concerning the roles of miRNAs in THCA is still limited. We therefore use a miRNA-target gene differential regulatory network (MGDRN) to identify key miRNAs and characterize their synergistic regulation in THCA. Both miRNA-target gene interactions from multiple databases and negative expression correlations between miRNA-target genes were used to characterize the interactions. Then, two regulatory networks involving normal and tumor conditions were constructed, respectively. The MGDRN was finally constructed using different interactions between the above two regulatory networks. By analyzing topological features of the MGDRN, four miRNAs (hsa-mir-152-3p, hsa-mir-148a, hsa-mir-130b and hsa-mir-15b) are identified as key miRNAs in THCA. Over-expression of mir-152-3p inhibited proliferation and colony formation of TPC-1 cells. Furthermore, mir-152-3p negatively regulated ERBB3 by binding to the 3'-UTR of ERBB3, and down-regulation of ERBB3 by small interfering (si)RNAs inhibited proliferation and colony formation of TPC-1 cells, indicating that mir-152-3p acted as an anti-tumor miRNA by negatively regulating ERBB3. Finally, two synergistically dysregulated modules were identified which may contribute to the initiation and progression of THCA. Overall, the results provided a better understanding of the molecular basis of THCA, and suggested novel treatment strategies for this cancer.

## Introduction

Micro(mi)RNAs are endogenous, single-stranded, non-coding RNAs (~ 22 nucleotides) that regulate gene expression by directly degrading mRNA or suppressing post-transcriptional protein translation by binding to the 3’ untranslated region (3’ -UTR) of the respective target mRNAs [1, 2]. The miRNAs have been reported to regulate ∼30% of the human genome, and are involved in many cellular processes such as cell proliferation [3], apoptosis [4], and development [5]. Abnormal miRNA functions may therefore affect multiple features of cells, resulting in complex pathological events including cancers. It has been confirmed that many miRNAs are tumor suppressors or oncogenes, and play important roles in the initiation, promotion and progression of various cancers [6].

Thyroid carcinoma (THCA) is one of the most common malignancies [7] and the fifth most frequent cancer in women, with increasing incidence[8]. Recent studies have emphasized the role of miRNA in THCA development [9]. Hong et al. found that miR-20b functioned as a tumor-suppressor in THCA by regulating the MAPK/ERK signaling pathway [10]. Using integrated miRNA and mRNA analyses, Liu et al. identified important miRNAs that were used to better understand the molecular mechanisms of THCA [11]. Most of these studies were based on differential expression of miRNAs. However, the expression of miRNAs is much lower than that of mRNA, and some important disease-related miRNAs do not differ in expression. Although the methods based on differential expression ignored the synergistic role of miRNAs, disease miRNAs tend to have more synergism, and regulate targets with the same or similar functions [12]. To avoid the ambiguities of slightly differentially expressed miRNAs, network based methods should use topological information to evaluate the importance of these molecules. At the same time, the differential regulatory network based method can identify both key molecules and key dysregulated relationships between disease and normal conditions, providing an opportunity to investigate the synergistic roles of miRNAs.

To further understand the possible role of miRNAs in THCA, we used a method based on a miRNA-gene differential regulatory network (MGDRN) to investigate the key miRNAs in THCA and to explore the synergistic role of miRNAs in this disorder. First, to improve the confidence of miRNA-target gene interactions, both miRNA-target gene interactions in multiple databases and negative expression correlations between miRNA-target genes were analyzed. Second, two regulatory networks were constructed involving normal or tumor conditions, respectively. The MGDRN was then constructed that included different interactions between the two regulatory networks. We then analyzed the topological features of the MGDRN and found key miRNAs involved in THCA. Experiments results showed that mir-152-3p acted as an anti-tumor miRNA by negatively regulating ERBB3. We further determined if these miRNAs synergistically dysregulated target genes at both gene level and pathway level. Our studies provided a useful tool to identify key miRNAs and dys-regulatory interactions in THCA that could help identify the molecular mechanism of this malignancy.

## Materials and method

### The expression data of genes and the miRNA of THCA

The RNA-seq datasets of genes and miRNA of THCA were downloaded from The Cancer Genome Atlas (TCGA) database (http://tcga-data.nci.nih.gov/), and then quantile-normalized and background-corrected at level three. Reads per kilobase of exon per million fragments mapped (RPKM) were used to describe the expression levels. The 463 cancer samples and 53 normal samples with matched gene expressions and miRNA profiles were extracted for analyses. For miRNA, the pre-miRNAs were converted to mature miRNAs (mat-miRNAs) based on the corresponding relationships between the pre-miRNAs and mat-miRNAs from the miRBase database [13].

### The miRNA-target gene interactions

The miRNA-target gene interactions were derived from seven databases including TargetScan [14], RNAhybrid [15], Rna22 [16], PicTar5 [17], mirBase [13], Miranda [18], and DIANA-microRNA. After redundancy processing, 289,470 miRNA-target interactions among 15,185 genes and 1,089 miRNAs were obtained.

### Construction of the MGDRN in THCA

We constructed the MGDRN by considering both predicted miRNA-target gene interactions and negative regulatory correlations. First, to improve the confidence of miRNA-target gene interactions, only interactions included in more than two databases were used. Then, differentially expressed genes were obtained by fold change (FC) method, and genes with a FC > 2 or < 0.5 were considered differential expressed. After logarithmic and absolute value transformation, the cutof is |log2(*FC*)| > 1. To identify THCA differentially regulated miRNA-target genes relationships, only miRNAs and differentially expressed genes in the miRNA-target gene interactions were extracted. Then, based on these THCA differentially regulated miRNA-target gene relationships, regulatory networks were constructed involving normal and tumor conditions, respectively. For each condition, the correlation values between each miRNA-gene interaction were calculated using Pearson's correlation coefficient (PCC) as follows: $PCC({miRNA}_{i,}{gene}_{i})=\frac{\sum_{i=1}^{n}\left(x_{i}-\bar{x})(y_{i}-\bar{y}\right)}{\sqrt{\sum_{i=1}^{n}{\left(x_{i}-\bar{x}\right)}^{2}{\left(y_{i}-\bar{y}\right)}^{2}}}$ (1), in which n represented sample numbers with both gene expression profiles and miRNA profiles; $x_{i}$ represented the expression value of genes in sample i, and $y_{i}$ represents expression value of miRNA in sample i; and $\bar{x}$ and $\bar{y}$ represented the mean expression values of the gene expression and miRNA expression in sample i, respectively. Because the miRNAs usually negatively regulated their genes, only the PCC between each miRNA-gene interaction less than 0 were remained.

Then, the two regulatory networks (normal and tumor) were compared and only the |PCC| with a difference > 0.2 and |log2(*FC*)| > 1 between tumor and normal were used to construct the MGDRN of the THCA. Finally, the MGDRN was constructed in which both the interactions between the miRNA-target and the expressions of nodes (miRNAs and targets) were differentially observed between tumor and normal samples.

### Topological measurement

For a given graph, G = (V, E), in which V represented the nodes, and E represented the edges. The degree measured how many edges connected to these nodes and reflected the interactions of these nodes with other nodes. For example, if there were n edges linked to a node, v, then the degree of node v was defined as:

Degree(v)=n;

Betweenness centrality measured the centrality of each node in a network. It was equal to the number of shortest paths from each node to all others that passed through this node, and it represented the amount of control that a node exerted over the interactions of other nodes in the network. The betweenness centrality of node v was defined as:

$$betweenness\;centrality(v)=\sum_{s\neq v\neq t}\frac{\sigma_{st}(v)}{\sigma_{st}};$$

where $\sigma_{st}$ was the total number of shortest paths from node s to node t and $\sigma_{st}(v)$ was the number of these paths that passed through node v.

The closeness centrality represented how close a node was to other nodes in the same network and was defined as the average mean path from this node to other nodes. The closeness centrality of node v was defined as:

$$Closeness\;centrality(v)=\frac{1}{\sum_{u}^{n}d(u,v)};$$

where d(u,v) represented the shortest distance between node u and the node v, and n represented the number of nodes in the network.

### Enrichment analyses

The gene ontology (GO) and Kyoto Encyclopedia of Genes and Genomes (KEGG) functional enrichment analyses were performed by DAVID tools (https://david.ncifcrf.gov/), which provided a comprehensive set of functional annotation and enrichment tools to understand the biological mechanisms of a gene set [19]. The biology process terms with P < 0.05 were considered statistically significant.

### Cell culture

The human cell lines TPC-1 was provided by the Chinese Academy of Medical. Then TPC-1 cells were cultured in Dulbecco’s modified Eagle medium with 10% fetal bovine serum (Invitrogen, Waltham, MA, USA), 50U/mL penicillin, and 50μg/ml streptomycin (Invitrogen). All cells were maintained at 37°C in a humidified incubator using 5% CO_2_.

### The small interfering (si)RNA and miRNA transfections

TPC-1 cells were seeded into 35mm plates at 24 hours before transfection. ERBB3 siRNA was used as the control siRNA, and was transfected using Lipofectamine^®^ 2000 (Invitrogen) with serum-free medium. At 5 hours after transfection, the medium was changed to complete medium, followed by 48 hours of culture.

The ERBB3 siRNA sequences uesed were as follow:

sense:5'-CCAAUACCAGACACUGUACUU-3’, and

antisense: 5'-GUACAGUGUCUGGUAUUGGUU- 3’.

TPC-1 cells were seed into 60 mm plates 24 hour prior transfection. 4ul of mir-152-3p-3p mimic or its corresponding negative control at 20uM (miR10000438-1-5, Ribobio, China) were transfected using lipofectamine 2000 (Invitrogen) for 48 h with serum-free medium according to experiments request.

### Antibodies and western blotting

Cells were lysed with RIPA lysis buffer containing a protease inhibitor cocktail (Roche, Basel, Switzerland). Equal amounts of protein (50 μg) were separated by 10% SDS-PAGE and transferred to a nitrocellulose membrane (Pall, Port Washington, NY, USA). After blocking, the blots were probed with primary antibodies to actin, ERBB3 (1: 200 dilution, Santa Cruz, Biotechnology, Santa Cruz, CA, USA), and caspase-3 (1: 500 dilution, Cell Signaling Technology, Danvers, MA, USA). After washing and incubating with rabbit or mouse secondary antibodies (1:10000 dilution; Cell Signaling Technology), the blots were visualized using the ECL reagent (GE Healthcare, Little Chalfont, UK).

### CCK-8 cell viability assay

TPC-1 cells were seeded into 96-well plates at a density of 2×10^3^ cells per well. Twenty-four hours later, they were transfected with the mir-152-3p-mimic or ERBB3-siRNA. After 48 hours, the cell viability was assessed using the Cell Counting Kit-8 (CCK-8; Dojindo, Tokyo, Japan).

### Clonogenic survival assay

The TPC-1 cells (8×10^2^) were counted and seeded into 6 cm dishes. After 48 hours of cell adherence, the cells weretransfected with the mir-152-3pmimic or ERBB3-siRNA. After 10 days of culture, the colonies were stained with 0.1% Crystal Violet in 20% methanol for 15 minutes. The samples were then photographed and the numbers of visible colonies were counted.

### Acridine orange/ethidium bromide (AO/EB) fluorescence staining

The TPC-1 cells were treated with the mir-152-3p-mimic or ERBB3-siRNA for 48 hours. The cells were then incubated with AO/EB mixing solution for 5 minutes (Solarbio Biotechnology, Beijing, China). Cellular morphological changes were examined by fluorescence microscopy at 200×. The percentage of apoptotic cells was calculated using the following formula: apoptotic rate (%) = number of apoptotic cells/ total number of cells counted.

### Luciferase reporter assay

The wildtype sequence of the 3'-UTR of ERBB3 (ERBB3-WT) and a mutant 3'-UTR of ERBB3 (ERBB3-Mut) were cloned into separate pMIR-REPORT luciferase vectors (Ambion, Thermo Fisher Scientific, Waltham, MA, USA). The HEK293 cells were seeded into six-well plates and co-transfected with the indicated reagents using Lipofectamine^®^ 2000 (Invitrogen) for 48 hours. The luciferase activity was assessed using the Dual-Luciferase-Reporter 1000 assay system (Promega, Madison, WI, USA). The Renilla activity was used for normalization.

### Data analysis

The data were obtained from at least there independent experiments, and were expressed as the mean ± standard deviation. The data were evaluated using the unpaired Student’s t test, and a value of P < 0.05 was considered to be statistically significant.

## Results

### Construction of the MGDRN

**Fig 1:**
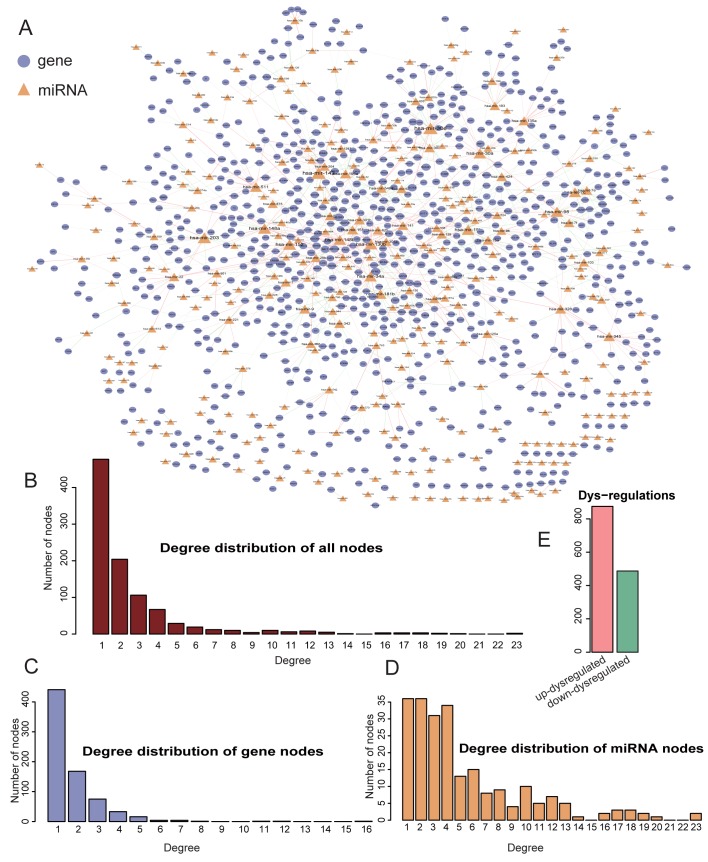
Topological features of the micro (mi)RNA-target gene differential regulatory network (MGDRN). **A.**Overview of the MGDRN; the purple and orange nodes represent mRNAs and miRNAs, respectively. The size of nodes represents the degrees of nodes in the network. The blue and pink edges in the MGDRM represent down-dysregulation and up-dysregulation involving tumor versus normal conditions, respectively. **B.** The node distribution of all nodes in the MGDRN. **C.** The node distribution of all mRNAs. **D.** The node distribution of all miRNAs. **E.** The number of down-dysregulated and up-dysregulated relationships in the MGDRN.

To construct the MGDRN, we first constructed a global miRNA-gene interaction model by using interactions included in more than two databases to obtain 282,053 interactions between 645 miRNAs and 14,591 genes. Differentially expressed genes were then obtained by the FC method, and genes with |log2(*FC*)| > 1 were considered differentially expressed. We obtained 3,872 differentially expressed genes. To identify the THCA differentially regulated miRNA-target gene relationships, only the differentially expressed genes and their regulating miRNAs in the miRNA-target genes interactions were extracted, resulting in 567 miRNA and 2759 genes remained. The negative regulatory relationships between miRNA-mRNA were also identified. Two regulatory networks were constructed involving both normal and tumor conditions by using the 2,759 differentially regulated genes and 567 miRNAs. For each condition, the correlation value between each miRNA-gene interaction was calculated using the Pearson's correlation coefficient (PCC), and only a PCC between each miRNA-gene interaction < 0 remained. The two regulatory networks were then compared, and only miRNA-gene interactions with a difference over 0.2 between tumor and normal remained. Finally, we constructed a MGDRN between the tumor and normal conditions by considering both predicted miRNA-target gene interactions and negative regulatory correlations of expression. The MGDRN of THCAs included 1,362 interactions between 304 miRNA and 826 genes. There were 875 up-dysregulated relationships (pink edges), compared with 487 down-dysregulated relationships (blue edges) (Fig 1A), suggesting that up-dysregulated relationships might play dominant roles in the progression of THCAs.

### Topological analyses of the MGDRN

**Fig 2:**
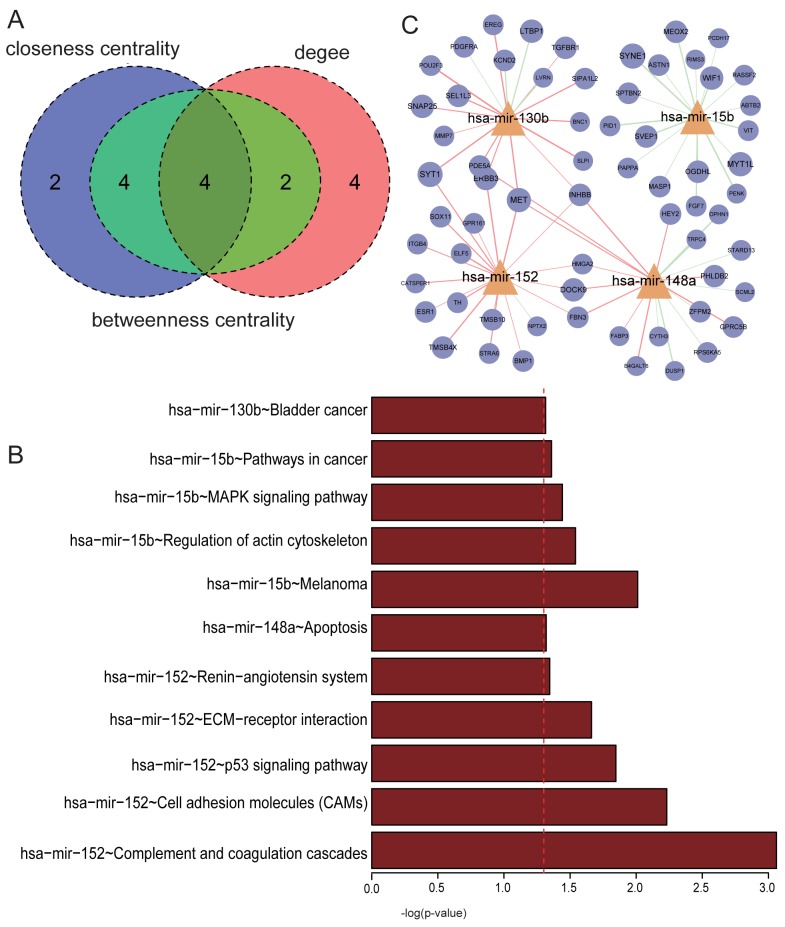
Topological analyses of the MGDRN. **A.**A Venn diagram of the top 10 maximum nodes in each dimension (with maximum degree, betweenness centrality, and closeness centrality). **B**. KEGG pathway enrichment analyses for dysregulated targets of four key miRNAs. The x-axis is -log_10_ of the P-value, and P< 0.05 was considered statistically significant. **C.** The subnetwork of four key miRNAs extracted from the MGDRN. Abbreviation is the same as in Fig 1.

We first analyzed the topological features of the MGDRN. For each node, the degree, betweenness centrality and closeness centrality were calculated. The distributions of all nodes, mRNA nodes, and miRNA nodes are shown in Fig 1B–1D. The degrees of all nodes and miRNAs ranged from 1 to 23, and the degrees of mRNA ranged from 1 to 16. A disperse distribution of miRNA nodes suggested that some miRNAs may be important regulators of multiple genes in THCA; while some miRNAs may be as specific regulators to regulate only a few genes. The topological features of all nodes’ of MGDRN were ranked, and Table 1 lists the top10 miRNAs on each dimension. Notably, four miRNAs (hsa-mir-152, hsa-mir-148a, hsa-mir-130b and hsa-mir-15b) were in the top ten in all topological features (Fig 2A and Table 1). It has been reported that hsa-mir-152 is repressed in endometrial cancer when compared to normal tissue, so it could be potential biomarker of endometrial cancer [20]. The hsa-mir-152-3p has also been reported to be specific for the follicular variant of papillary thyroid cancers [21]. To investigate the biological mechanisms of these miRNAs in the MGDRN, GO function and KEGG pathway enrichment analyses were performed for each miRNA by using their dysregulated target genes in MGDRN (S1–S4 Tables and Fig 2B). As shown in Fig 2B, dysregulated target genes of hsa-mir-152 were enriched in most processes including complement and coagulation cascades, cell adhesion molecules, p53 signaling pathway, ECM-receptor interaction and the renin-angiotensin system. The hsa-mir-148a was involved in the apoptosis pathway; the hsa-mir-15b participated inregulation of the actin cytoskeleton, the MAPK signaling pathway and pathways in cancer; and the hsa-mir-130b was enriched in the bladder cancer pathway. Most of the significant KEGG terms of these miRNAs focused on cancer-related processes, suggesting these miRNAs play important roles in cancer progression and may serve as key regulators in THCA.

**Table 1:** The top 10 miRNAs in degree, betweennesscentrality and closenesscentrality.

name	Degree	name	Closeness Centrality	name	Betweenness Centrality
hsa-mir-30e	23	hsa-mir-152-3p	0.206728	hsa-mir-15b	0.11699
hsa-mir-142	23	hsa-mir-15b	0.204809	hsa-mir-130b	0.097815
hsa-mir-203	20	hsa-mir-130b	0.203692	hsa-mir-152	0.095341
hsa-mir-152-3p	19	hsa-mir-301b	0.201829	hsa-mir-142	0.092238
hsa-mir-148a	19	hsa-mir-148b	0.199179	hsa-mir-148b	0.082839
hsa-mir-130b	18	hsa-mir-148a	0.19636	hsa-mir-30e	0.080211
hsa-mir-98	18	hsa-mir-181b	0.196043	hsa-mir-148a	0.067307
hsa-mir-15b	18	hsa-mir-29b	0.195097	hsa-mir-98	0.067228
hsa-mir-30a	17	hsa-mir-30e	0.194006	hsa-mir-511	0.060459
hsa-mir-34a	17	hsa-mir-142	0.19285	hsa-mir-328	0.05879

To further investigate the mechanism of the four miRNAs, the miRNAs and their dysregulated target relations were extracted from the MGDRN. The results showed that the majority of PCCs between has-mir-130b, has-mir-152, and has-mir-148a and their targets were up-regulated in tumor conditions, while the PCCs between has-mir-15b and its targets were down-regulated in tumor conditions (Fig 2C). In addition, these miRNAs shared some target genes, suggesting that they were synergistically dysregulated in THCA. Notably, all of PCCs between has-mir-152 and its targets were up-regulated in tumors (Fig 2C), indicating that the negatively regulated effect of mir-152 was reduced in tumors. Thus, mir-152 may be a potential tumor-suppressing miRNA [22]. To confirm the isoform of mir-152, we used TargetScan bioinformatics tools and found that miR-152-3p could target ERBB3, so miR-152-3p was used for the following experiments.

### Over-expression of mir-152-3p inhibits cell proliferation and colony formation of TPC-1 cells

**Fig 3:**
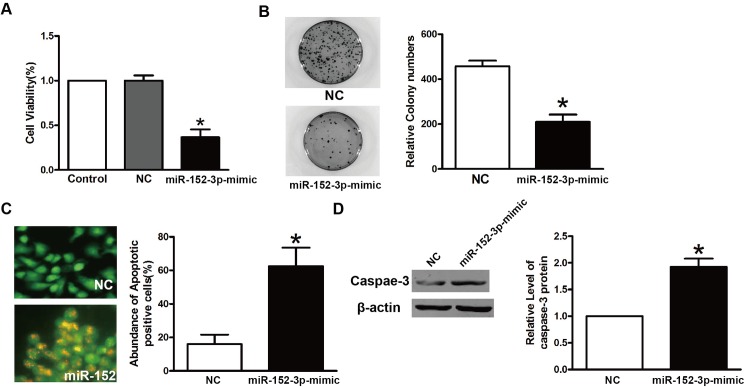
The mir-152-3p inhibits proliferation and colony formation of TPC-1 cells. **A.** The MTT assay of TPC-1 cells infected with the NC or mir-152-3p mimic. *P<0.05. **B.** The colony formation assay of TPC-1 cells infected with the NC or mir-152-3p mimic. top, representative images. Bottom(graph), colony numbers from three independent experiments. *P<0.05. **C**. The acridine range/ethidium bromide ratio was determined to characterize the apoptosis of TPC-1 cells. **D**. The expression of caspase-3 in TPC-1 cells after treatment with the NC or mir-152-3pmimic was determined by western blots. *P<0.05.

To further test the tumor-suppressing effects of mir-152-3p on THCAs, we investigated the effects of mir-152-3p on cell proliferation and colony formation in TPC-1 cells transfected with the mir-152-3p-mimic or NC. The CCK-8 assay showed that TPC-1 cells transfected with the mir-152-3p-mimic grew slower than their NC-transfected counterparts (Fig 3A). The colony formation assay showed that TPC-1 cells transfected with mir-152-3p-mimic showed fewer colonies than their NC transfected counterparts (Fig 3B). The AO/EB stainding showed that after treatment with the mir-152-3p-mimic, the TPC-1 cells underwent apoptosis (Fig 3C). The protein levels of caspase-3 also showed that the cells transfected with the mir-152-3pmimic were more upregulated than those of the NC group.

### ERBB3 is a direct target of mir-152-3p

**Fig 4:**
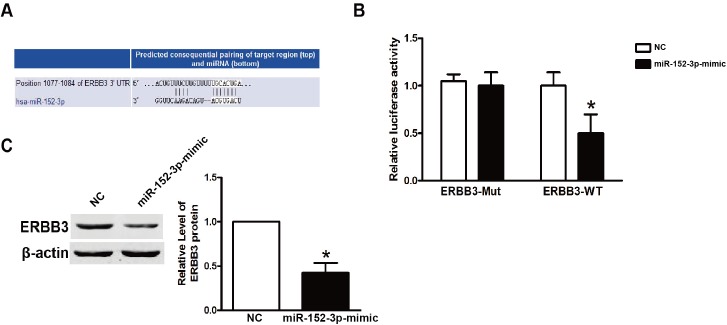
The mir-152-3p negatively regulates ERBB3 by binding to the 3'-UTR of ERBB3. **A.** Putative mir-152-3p-binding site at the 3'-UTR of ERBB3(TargetScan). **B.**The luciferase assay of HEK293T cells. The relative luciferase activity was determined after the described reporter constructs (pMIR-ERBB3-WT or pMIR-ERBB3-Mut) were co-transfected with the NC or mir-152-3p mimic into HEK293T cells. The luciferase activity was normalized to Renilla luciferase activity. Columns, mean; bars, standard deviation. ^**^P<0.01. **C.** The mir-152-3p negatively regulates protein expression of ERBB3 in TPC-1 cells. β-Actin was used as an internal control for ERBB3.

To characterize the molecular mechanism of growth inhibition by mir-152-3p, we searched for genes containing potential mir-152-3p recognition sites in their 3'-UTRs using the TargetScan bioinformatics tools. which showed that the oncogene, ERBB3, was a putative target of mir-152-3p (Fig 4A). Some miRNAs have been reported to have an anti-tumor effect that involved negative regulation ERBB3 [23]. To confirm the specificity of mir-152-3p, the wild type sequence of the ERBB3 3'-UTR (ERBB3-WT) and a mutant ERBB3 3'-UTR (ERBB3-Mut) were inserted into the pMIR-REPORT luciferase vector. After co-transefection of the indicated reagents described in Fig 4B, mir-152-3p significantly reduced the luciferase activity of ERBB3-WT, while the ERBB3-Mut did not show any differences. In addition, we performed immunoblot analyses of NC and the mir-152-3p-mimic-transfected cell extracts using anti-ERBB3-specific antibody. Enforced expression of mir-152-3p resulted in a potent downregulation of ERBB3 protein levels in TPC-1 cells (Fig 4C).

### Downregulation of ERBB3 by specific siRNAs inhibits proliferation and colony formation of TPC-1 cells

**Fig 5:**
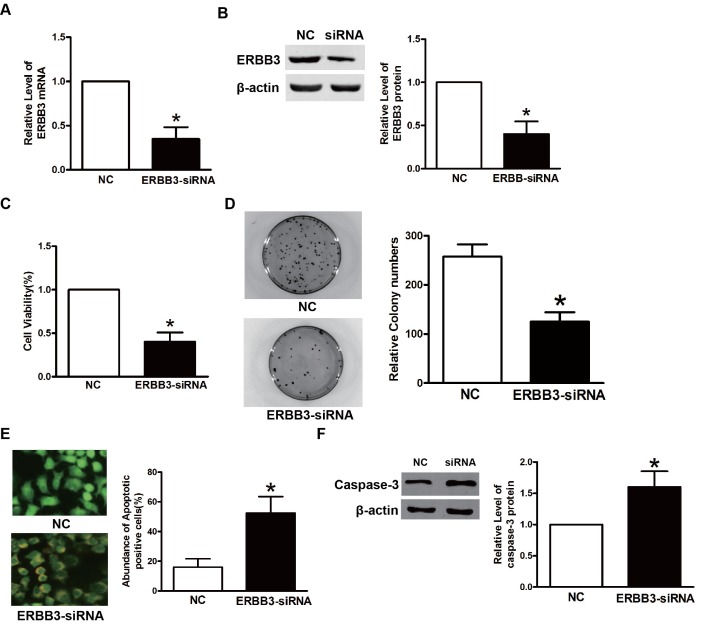
Downregulation of ERBB3 by specific siRNAs inhibits proliferation and colony formation of TPC-1 cells. **A.** The mRNA expression of ERBB3 in TPC-1 cells transfected with ERBB3-siRNA or NC. β-Actin served as an internal control. *P<0.05. **B.** The protein expression of ERBB3 in TPC-1 cells transfected with ERBB3-siRNA or NC. β-Actin served as an internal control. **C.** The MTT assay TPC-1 cells transfected with ERBB3-siRNA or NC. *P<0.05. **D**. The colony formation assay of TPC-1 cells transfected with ERBB3-siRNA or NC. *P<0.05, **E.** The acridine range/ethidium bromide ratio was used to determine the apoptosis of TPC-1 cells. **F.** The expression of caspase-3 in TPC-1 cells after treatment with NC or ERBB3-siRNA.^*^P<0.05.

Because we showed that ERBB3 was a target of mir-152-3p, we postulated that mir-152-3p inhibited TPC-1 cells by downregulating ERBB3. To confirm that downregulation of ERBB3 is crucial for the inhibitory effects on TPC-1 cells, specific siRNA against ERBB3 was used to silence ERBB3. As shown in Fig 5A and 5B, the si-ERBB3 significantly reduced the expression of ERBB3 mRNA and protein. CCK-8, AO/EB, and colony formation assays showed that si-ERBB3 inhibited TPC-1 cell proliferation and colony formation (Fig 5C–5E). Furthermore, the immunoblots showed elevated protein levels of caspase-3, suggesting that silencing of ERBB3 resulted in increased apoptosis (Fig 5F). Together, these results showed thatmir-152-3p induced the inhibition of TPC-1 cell proliferation and induced apoptosis of these cells.

### The miRNAs involved synergistically and dysregulated pathways in THCA

**Fig 6:**
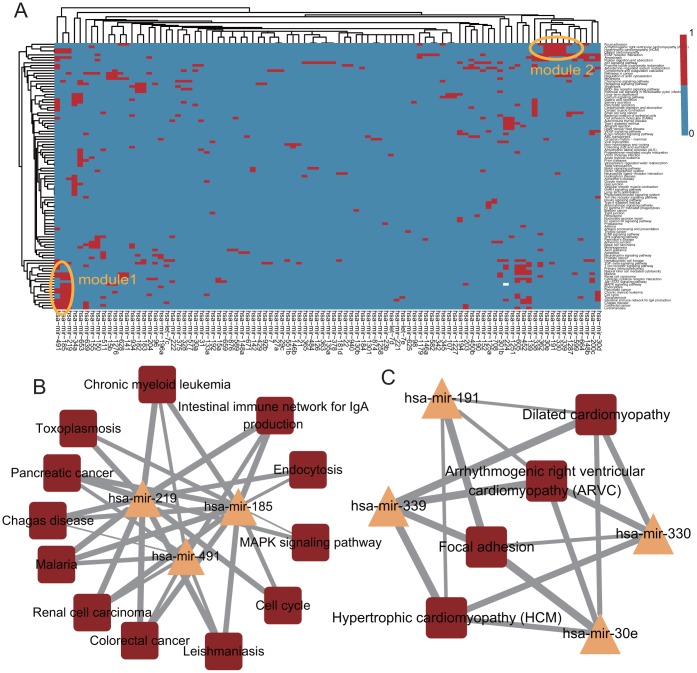
The analyses of synergistically dysregulated pathways by miRNAs. **A. A** heatmap of the synergistically dysregulated pathways by miRNAs; bi-directional hierarchical clustering was performed using the R package. The two yellow ellipses represent module 1 and module 2, respectively. **B**. The sub-network of module 1.**C**. The sub network of module 2.

Previous studies reported that multiple miRNAs synergistically controlled individual genes [12], so we determined if these miRNAs synergistically dysregulated target genes in the MGDRN. A bi-directional hierarchical clustering was performed on the MGDRN. Although some miRNAs dysregulated multiple genes in THCA, there was no significant modularity phenomenon (S1 Fig), suggesting that these miRNAs did not synergistically dysregulate genes in THCAs. Furthermore, we investigated whether miRNAs synergistically dysregulated genes on a pathway level. First, KEGG pathway enrichment analyses were performed to identify miRNA dysregulated pathways using their target dysregulated genes in the MGDRN. Similarly, bi-directional hierarchical clustering was performed on miRNA and dysregulated pathways. The clustered miRNAs tend to dysregulate similar biological functions. We found that a single miRNA could dysregulate multiple pathways and that a single pathway could be synergistically dysregulated by multiple miRNAs (Fig 6A). The results showed obvious modularity in the heatmap plot (Fig 6A), suggesting that miRNAs synergistically dysregulated genes on the pathway level in THCA. Two modules were discovered in the heatmap plot. Module 1 showed that three miRNAs (hsa-mir-491, hsa-mir-185 and hsa-mir-219) synergistically dysregulated 12 functional pathways (Fig 6B). Most of these pathways involved cancer or cancer related (i.e., the MAPK signaling pathway) pathways. Furthermore, it has been reported that hsa-mir-219 inhibited tumor size and cancer cell proliferation, suggesting that it was a negative regulator of tumor development [24]. Forced expression of miR-219-5p suppressed PTC cell proliferation and migration and promoted apoptosis[25]. The miR-491 regulated the proliferation and apoptosis of CD8(+) T cells that could be a novel target for antitumour immunotherapy [26]. The miRNA-185 suppressesed proliferation, invasion, migration, and tumorigenicity of human prostate cancer cells by targeting the androgen receptor [27]. These three miRNAs may therefore play crucial roles by synergistically dysregulating these cancer related pathways in THCAs. Module 2 showed that four miRNAs (hsa-mir-30e, hsa-mir-191, hsa-mir-330 and has-mir-339) synergistically dysregulated four functional pathways (Fig 6C). In non-alcoholic fatty liver disease, hsa-miR-330 has been reported to modulate focal adhesion by targeting VEGFA and CDC42 [28]. Focal adhesion is also an import pathway in THCA [29], suggesting hsa-miR-330 may be involved in this process in THCAs. The hsa-mir-191 is plays an important role in many cancers including ovarian endometriosis and osteosarcoma [30, 31]. Notably, the other three pathways involved cardiovascular disease pathways, indicating underlying molecular interactions between THCA and cardiovascular disease. Klein et al. suggested that the risk of cardiovascular and all-cause mortality is increased in patients with THCA, independent of age, sex, and cardiovascular risk factors and that a lower thyroid stimulating hormone level may have been responsible for this increased mortality[32].

## Discussion

In the present study, we used a MGDRN to identify key miRNAs and to explore the synergistic roles of these miRNAs in THCAs. First, both miRNA-targeted gene interactions in multiple databases and negative expression correlations between miRNA-target gene were used to validate miRNA-target gene interactions. Second, two regulatory networks were constructed involved normal and tumor condition, respectively. Finally, the MGDRN was constructed by interactions that differed between the above two regulatory networks. The MGDRN of THCA included 1,130 nodes (304 miRNAs and 826 genes) and 1,362 edges. The 875 up-dysregulated relationships (pink edges) and 487 down-dysregulated relationships (blue edges) involving tumor conditions suggested that up-dysregulated relationships played a dominant role in progression of THCAs. By analyzing topological features (degree, betweenness centrality and closeness centrality) of the MGDRN, four miRNAs (hsa-mir-152-3p, hsa-mir-148a, hsa-mir-130b and hsa-mir-15b) are identified as key miRNAs in THCAs. Overall, the results showed that mir-152-3p induced an anti-tumor effect by negatively regulating ERBB3.

We further explored if miRNAs synergistically dysregulated target genes in the MGDRN. We found that miRNAs synergistically dysregulated genes on the pathway level rather than on the gene level in THCAs. Two synergistically dysregulated modules were identified that could contribute to the initiation and progression of THCA.

The success of this study could be attributed to two reasons. First, the method we used to construct differential regulatory networks could identify dysregulated molecules and dysregulated miRNA-target interactions in THCAs at the same time, making it possible to characterize the synergistic roles of miRNAs. We also chose different cutoffs of 7 miRNA-mRNA databases (intersection = 3,4,5,6 and 7, respectively; see S5 Table). We found that when cutoff = 3, the miRNAs, mRNAs and their relationship in the MGDRN dramatically decreased compared with cutoff = 2. When cutoff = 7, there was no miRNA-mRNA relationship predicted. So we chose seven algorithms and a cutoff of >2 to guarantee a relatively higher sensitivity and accuracy. We then used topological information to evaluate the importance of molecules to avoid a problem with slightly differentially expressed miRNAs. Second, in construction of the MGDRN, both miRNA-target gene interactions in multiple databases and negative expression correlations between miRNA-target genes were used to improve the confidence of miRNA-target gene interactions.

In summary, we identified putative miRNAs and modules that were involved in THCAs by using a differential regulatory network, which provided a better understanding of the molecular basis of THCA.

## Supporting information

S1 Fig Heatmap of the genes dys-regulated by miRNAs; bi-directional hierarchical clustering was performed using the R package. (PDF)

S1 Table The gene ontology (GO) enrichment result of the miRNA has-mir-130. (TXT)

S2 Table The gene ontology (GO) enrichment result of the lncRNA has-mir-148a. (TXT)

S3 Table The gene ontology (GO) enrichment result of the miRNA has-mir-152. (TXT)

S4 Table The gene ontology (GO) enrichment result of the miRNA has-mir-15b. (TXT)

S5 Table The number of mRNA, miRNA and edge in the MGDRN. (DOCX)
