# Supplementary figures and images for: Construction and analyses of the microRNA-target gene differential regulatory network in thyroid carcinoma
Source: PLoS One. 2017 Jun 1;12(6):e0178331. doi: 10.1371/journal.pone.0178331 (PMC5453480; doi:10.1371/journal.pone.0178331)

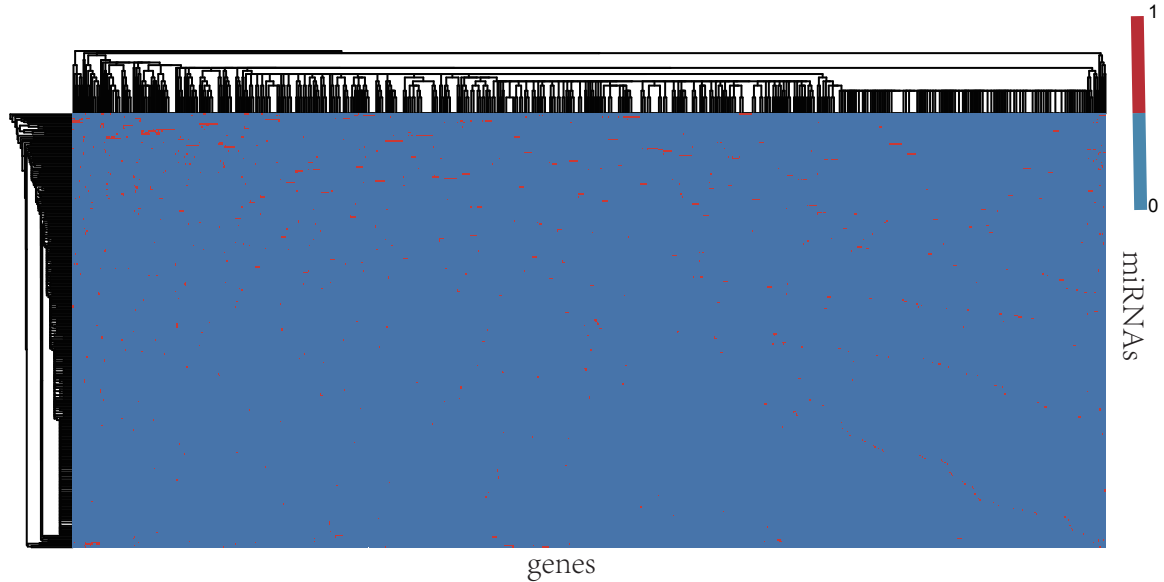

Supplement: S1 Fig — (PDF) [file pone.0178331.s001.pdf]
