# Supplementary material for: Construction and analyses of the microRNA-target gene differential regulatory network in thyroid carcinoma
Source: PLoS One. 2017 Jun 1;12(6):e0178331. doi: 10.1371/journal.pone.0178331 (PMC5453480; doi:10.1371/journal.pone.0178331)
Supplement: S5 Table — (DOCX) [file pone.0178331.s006.docx]

Table S5 The number of mRNA, miRNA and edge in the MGDRN

| Cutoff | Number of mRNA | Number of miRNA | Number of edge |
| --- | --- | --- | --- |
| 2 | 826 | 304 | 1362 |
| 3 | 215 | 138 | 274 |
| 4 | 45 | 40 | 52 |
| 5 | 12 | 12 | 13 |
| 6 | 1 | 1 | 1 |
| 7 | 0 | 0 | 0 |
